# Supplementary material for: Evolution and Efficiency Assessment of Pesticide and Fertiliser Inputs to Cultivated Land in China
Source: Int J Environ Res Public Health. 2021 Apr 4;18(7):3771. doi: 10.3390/ijerph18073771 (PMC8038477; doi:10.3390/ijerph18073771)
Supplement: Supplementary file 1 [file ijerph-18-03771-s001.pdf]

Table S1. Existing studies on factors influencing fertilizer and pesticide application.

Influencing Factors	Author	Paper Name	Journal Name	Main Content
Primary industry value added	WANG Yi-Jie,DI Fei,XIN Ling	The status and problems of grain production in the main grain production areas of China and policy suggestions [9]	Research of Agricultural Modernization	Wang Yijie et al. used a comparative study analysis to point out that excessive use of chemical fertilizers and pesticides can cause variability in production levels in major grain producing areas and thus affect grain yields and other problems.
	Yang Zonghui, Cai Hongyi, Qin Cheng, Liu Heguang	Analysis on the Spatial and Temporal Pattern of China's Grain Production and Its Influencing Factors [10]	Journal of Agricultural Science and Technology丨J Agric Sci Tech China	Yang Zonghui et al. used the SDM model to empirically study the main influencing factors of grain production in China's provinces and concluded that the amount of grain production in China's provinces is positively related to the size of the role of the actual watering area per unit and the amount of chemical fertilizers.
	Wang Zeyu, Li Gucheng, Zhou Xiaosi	Structure change of rural labor force,grain production and fertilizer using efficiency promotion;An empirical study based on stochastic frontier production function and Tobit model [11]	Journal of China Agricultural University	Wang Zeyu et a l. analyzed the positive relationship between agricultural labor structure and fertilizer application efficiency based on panel data using Tobit model study, which contributes to the production of food.
	Yang Xinghong, Zhang Fanfan, Zhang Qinan	Research on Measurement of Contribution Rate of Essential Factors in Major Grain Producing Areas [12]	Price:Theory & Practice	Yang Xinghong et al. analyzed the relationship between fertilizer application and the contribution of grain yield growth by C-D production function and concluded that fertilizer application has a tendency to have a weakening effect on grain yield increase.
	Guo Sihua, Ji Kaiwen	Measurement of Rice Production Efficiency in Jiangxi and Analysis of Its Influencing Factors [13]	Journal of Jiangxi University of Finance and Economics	Guo S. et al. empirically investigated the factors at play in the production efficiency of early indica rice using the Tobit model and concluded that factors such as fertilizers and pesticides suffer from real losses in terms of inputs, which are not conducive to increased grain output.
	Xu Dongting	Grey correlation analysis of factors influencing grain yield in Jiangsu Province [14]	Grain Science and Technology and Economy	Based on the grain data of Jiangsu Province from 2000 to 2018, Dongting Xu used a gray correlation model to analyze the dynamics of six factors influencing grain yield in Jiangsu Province, and concluded that labor input, fertilizer application and pesticide application in grain production had a more significant effect on grain yield.
Rural business scale	Tian Yun, Zhang Junbiao, He Ke, etc.	Analysis of farmers' agricultural low-carbon production behaviors and their influencing factors - taking fertilizer application and pesticide use as examples. [15]	China Rural Survey	Tian Yun et al. found that farmers who were male, had been farming for a longer period of time, and had smaller arable areas tended to apply pesticides and fertilizers in lower amounts.
	Wu Y Y, Xi X C, Tang X, et al.	Policy distortions, farm size, and the overuse of agricultural chemicals in China. [16]	Proceedings of the National Academy of Sciences of the United States of America.	Based on a survey of rural households in China, Wu et al. found that the scale of operation was an important factor influencing the intensity of pesticide application.
	Zhang Shuxian, Chen Meiqiu,	A study on the ecological farming behavior of farmers with different operation scales:	Ecological Economy	Based on the application of pesticides and fertilizers by farmers, Zhang Shuxian et al. used the OrderedProbit model to provide an in-depth analysis of the ecological farming behavior of farmers of different operation

	Kuang Fo Yuan	an example of pesticide and fertilizer application. [17]		scales and its influencing factors, and found that there were differences in the choice of pesticides by farmers of different operation scales.
	Ying Sun	The Impact of Labor Mobility and Land Scale Management on the Development of Agricultural Modernization: A Case of County Panel Data in Jiangsu Province. [18]	Rural Economy and Science-Technology	The results showed that the fertilizer and pesticide use per unit sown area was significantly and negatively correlated with the average household sown area, which indicated that the expansion of the average household sown area would help to reduce the use of fertilizer and pesticide.
	Jingjing Gao, Chao Peng, Qinghua Shi	A study on high fertilizer use and fertilizer application behavior of smallholder farmers in China--findings based on data from fixed rural observation sites nationwide from 1995 to 2016. [19]	Management World	The problem of irrational fertilizer application is closely related to the production scale of farmers, and factors such as the small size of the average household land and the large degree of fragmentation determined by the national reality are important reasons for the higher fertilizer application by Chinese farmers than the world average.
	Sun Fangling	Study on the reduction of chemical fertilizers for food and agriculture in Henan Province based on the scale of land operation--Xihua County as an example. [20]	Henan University of Economics and Law	This paper analyzes the research data of wheat growers in Xihua County, Henan Province, to derive the relationship between land operation scale and fertilizer application, and further measures the optimum planting scale for reasonable fertilizer application.
	Ma Xianlei, Che Xuchao, Li Na, Tang Liang	Did arable land transfer and large-scale management improve the agricultural environment? -- A test based on the effect of arable land use behavior on agro-environmental efficiency. [21]	China Land Science	The scale of arable land use can negatively affect agro-environmental efficiency by changing the intensity of fertilizer and machinery inputs.
	LiYunju,FredrichKahrl,PanJianjun,David Roland-Holst,SuYufang,Andreas Wilkes,XuJianchu.	Fertilizer use patterns in Yunnan Province,China:Implicationsfor agriculturalandenvironmental policy. [22]	Agricultural Systems	JIANGL and LIZH concluded that a decrease in land area per capita was associated with an increase in fertilizer application intensity. Farm size was negatively correlated with fertilizer inputs, i.e., increasing the size of land operations facilitated the reduction of fertilizer inputs.
	Zou Wei, Zhang Xiaoyuan	Effects of land management scale on fertilizer use efficiency: Taking Jiangsu as an example. [23]	Resources Science	Farmers' business situation before land expansion also influences their fertilizer application behavior.
	Shi Changliang, Zhang Yi, Guo Yan, and Zhu Junfeng.	The effect of arable land fine fragmentation on the efficiency of fertilizer use by farmers. [24]	Journal of Natural Resources	The fine fragmentation of arable land has a significant negative effect on the efficiency of fertilizer use by farmers and is an important cause of inefficient fertilizer use by farmers.
Aggregate Producer Price Index for Agricultural Products	Chun Xiao	Analysis of cotton policy, price fluctuation and production investment behavior of cotton farmers--an example from Xinjiang cotton region [25]	Price：Thecory & Practice	Wang Chunxiao took Xinjiang cotton region as an example, and the results showed that in the main cotton producing areas, the increase of cotton price was positively related to the scale of cotton farmers' production investment, including the increase of planting area and material inputs such as fertilizers and pesticides. However, when the cotton price decreases, cotton farmers do not reduce the production investment scale by reducing planting area, but choose to reduce fertilizer, pesticide and other material inputs in order to obtain relatively high net profit.

Xingxiao Ma	Impact of Agricultural Product Prices on Farmers' Production Investment - An Empirical Analysis Based on Interprovincial Panel Data [26]	Tianjin University of Commerce	The price levels of production factors such as fertilizers, pesticides, agricultural machinery and hired labor have less significant and less significant effects on various types of capital investments of farm households than the prices of agricultural products. When making production factor input decisions, their prices are not an important consideration, and they are generally based on years of farming experience with changes in expected income.
Liu Yong, Wei Junying	An empirical analysis of the effect of agricultural product prices on fertilizer use [27]	Rural Economy and Science-Technology	The study found a positive relationship between agricultural commodity prices and fertilizer application, while other control variables fertilizer prices, fertilizer output rates, and policy variables showed negative relationships with fertilizer application.
Wang Meibu, Tian Minghua	Interrelationship between fertilizer application intensity and grain trade in China based on VECM model [28]	Ecological Economy	Wang, Mei-rabbit, and Tian, Minghua found that the price of agricultural products and farmers' income can make the fertilizer application intensity decrease, while the total import and export of food and the price of agricultural production materials can make the fertilizer application intensity increase.
